# Treatment of Postoperative Instability Following Total Knee Arthroplasty in Patients With Parkinson’s Disease

**DOI:** 10.1016/j.artd.2023.101273

**Published:** 2023-12-28

**Authors:** Catherine M. Call, Brian J. McGrory, Erica A. Thompson, Lydia G. Sommer, Thomas S. Savadove

**Affiliations:** a Tufts University School of Medicine, Boston, MA, USA; b Division of Joint Replacement Surgery, Maine Medical Center, Portland, ME, USA; c Department of Rehabilitation, Maine Medical Center, Portland, ME, USA; d Department of Physical Medicine and Rehabilitation, Maine Medical Center, Portland, ME, USA

**Keywords:** Total knee replacement (TKA), Parkinson’s disease (PD), Dislocation, Instability, Botulinum toxin A, Neurodegenerative disorder

## Abstract

Acute postoperative posterior total knee arthroplasty (TKA) dislocation is rare in primary surgery but has been associated with Parkinson’s disease (PD). We present a 77-year-old woman with knee arthritis and PD who sustained an acute, recurrent TKA posterior dislocation, recalcitrant to polyethylene upsizing. Transient stability was obtained for a period of 1 year after postoperative hamstring injection with botulinum toxin A and short-term immobilization. Spontaneous instability recurred after 1 year, and stability was obtained with revision to a more constrained construct and has been monitored over a period of 2 years. This is the first report demonstrating the use of botulinum toxin A for acute posterior TKA instability associated with PD. We endorse the necessity of increased constraint to maintain long-term stability in patients with Parkinson’s disease.

## Introduction

Total knee arthroplasty (TKA) is normally an exceptionally successful surgery that relieves pain and restores range of motion (ROM), stability, and function in patients with end-stage arthritis. Joint replacement outcomes have been reported to be less predictable in patients with a concomitant diagnosis of Parkinson’s disease (PD), a neurodegenerative disorder [1,2]. The incidence of perioperative complications is likely also higher in patients with PD who undergo TKA because of concomitant neurologic abnormalities including cognitive issues, swallowing disorders, muscle and capsule contractures, tremors, muscle spasms, rigidity, and poor balance [1,3,4].

We present the case of a 77-year-old woman with knee arthritis, as well as well-controlled mild (modified Hoehn and Yahr [5] stage 2.5) PD at initial presentation, who sustained acute instability after a posterior stabilized (PS) TKA, which recurred despite polyethylene revision and upsizing. Successful stability was obtained for a period of 1 year after postoperative hamstring injection with botulinum toxin A (BoNTA) and short-term immobilization, but instability recurred 1 year after revision. She underwent a repeat revision to a more constrained implant followed by hamstring injection of BoNTA. Successful stability was obtained and has been monitored over a period of 2 years. To our knowledge, this is the first report demonstrating the use of BoNTA in joint surgery patients with PD and a potential role for planned postoperative BoNTA injection to aid in TKA recovery in select patient populations.

## Case history

### Index procedure

A 77-year-old female who provided written informed consent for us to report her case was admitted with right knee osteoarthritis for an elective TKA. Her past medical history includes mild severity PD (modified Hoehn and Yahr [5] (Table 1) stage 2.5 diagnosed 3 years prior to presentation and nonprogressive in severity since diagnosis, managed with 50 mg-200 mg of carbidopa–levodopa 4 times each day); osteoarthritis (status post left cruciate-retaining cemented TKA 18 months prior for milder deformity, without complication); anxiety; spinal stenosis; scoliosis; treated sleep apnea; asthma and restless leg syndrome, at a body mass index of 38. The patient utilized a cane intermittently at baseline and had difficulty walking on uneven ground and climbing stairs. She could walk for 5-15 minutes without pain. She was not taking any preoperative pain medications or antispasmodics.

**Table 1:** Modified Hoehn and Yahr scale.

Stage	Description
1	Mild unilateral symptoms that do not interfere with daily activities.
1.5	Unilateral symptoms with axial involvement.
2	Symptoms present bilaterally. Balance remains intact.
2.5	Mild bilateral disease with recovery on pull test.
3	Symptoms are mild to moderate bilaterally, with balance impairment and slowness. Physically independent.
4	Symptoms are severe and limiting. Patient is still able to stand unassisted. No longer independent with daily activities.
5	Patient is wheelchair-bound or bedridden unless assisted, requires continuous care.

**Figure 1:**
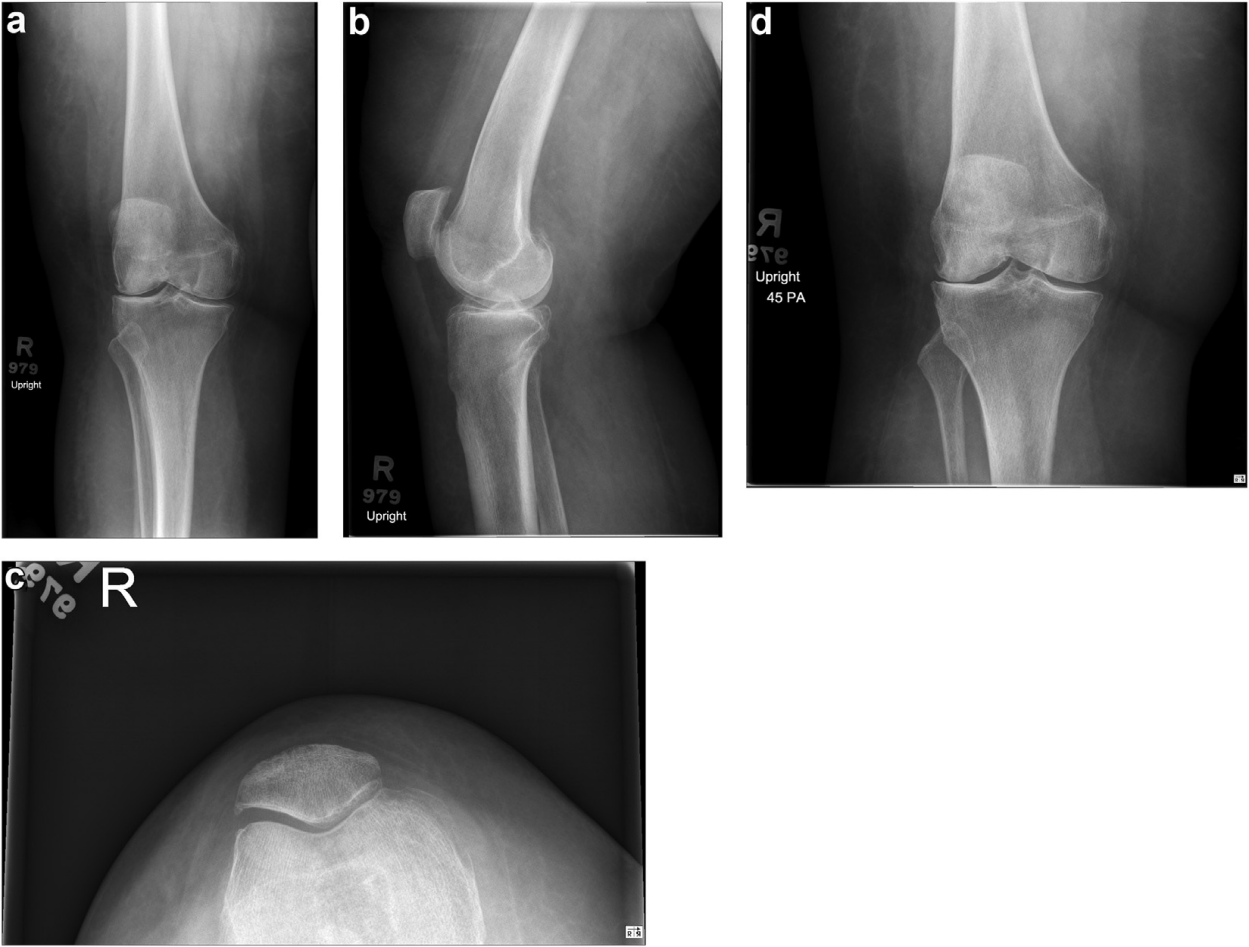
(a) Anteroposterior (AP), (b) lateral, (c) Merchant, and (d) Posteroanterior (PA) flexion knee radiographs demonstrate right tri-compartmental arthritis with valgus disposition.

Her preoperative right knee ROM was from 15 to 110 degrees of flexion, without instability, as indicated by medial/lateral and anterior/posterior stress testing at 15, 45, and 90 degrees of flexion. Mild hand tremor and upper extremity cogwheeling rigidity were present on exam, but there was no marked hamstring spasm. Anteroposterior, posteroanterior flexion, lateral, and merchant knee radiographs demonstrated tricompartmental arthritis with valgus alignment (Fig. 1).

She underwent right-cemented TKA (NexGen LPS, Zimmer Biomet, Warsaw, IN) with a PS polyethylene on the day of admission using a medial parapatellar approach. Spinal anesthesia was administered, in addition to a postoperative adductor canal nerve block utilizing ultrasound guidance. A gap-balancing technique was utilized: the femur was a size C, tibia was size 2, and a 12 mm standard tibial component was utilized; the patella was resurfaced. Full painless ROM with excellent stability through arc of motion was achieved from 0 to 130 degrees of flexion.

Upon arrival to the inpatient unit, the patient was mobilized by a physical therapist (PT) using a stand pivot transfer from bed to commode. It was documented that she was not confident with this transfer and hesitant to weight-bear through her operative leg. Right knee ROM was noted to be 20-60 degrees of flexion, and her quadriceps strength was grossly 3/5.

The patient experienced difficulty with pain control overnight and on her first postoperative day (POD). She was only able to stand briefly at the edge of bed on POD 1 secondary to pain and weakness, as documented by PT; there was no deformity noted at this time. There was no injury or specific event that was noted where her pain increased dramatically, and the knee was not noticed to be dislocated at this time.

**Figure 2:**
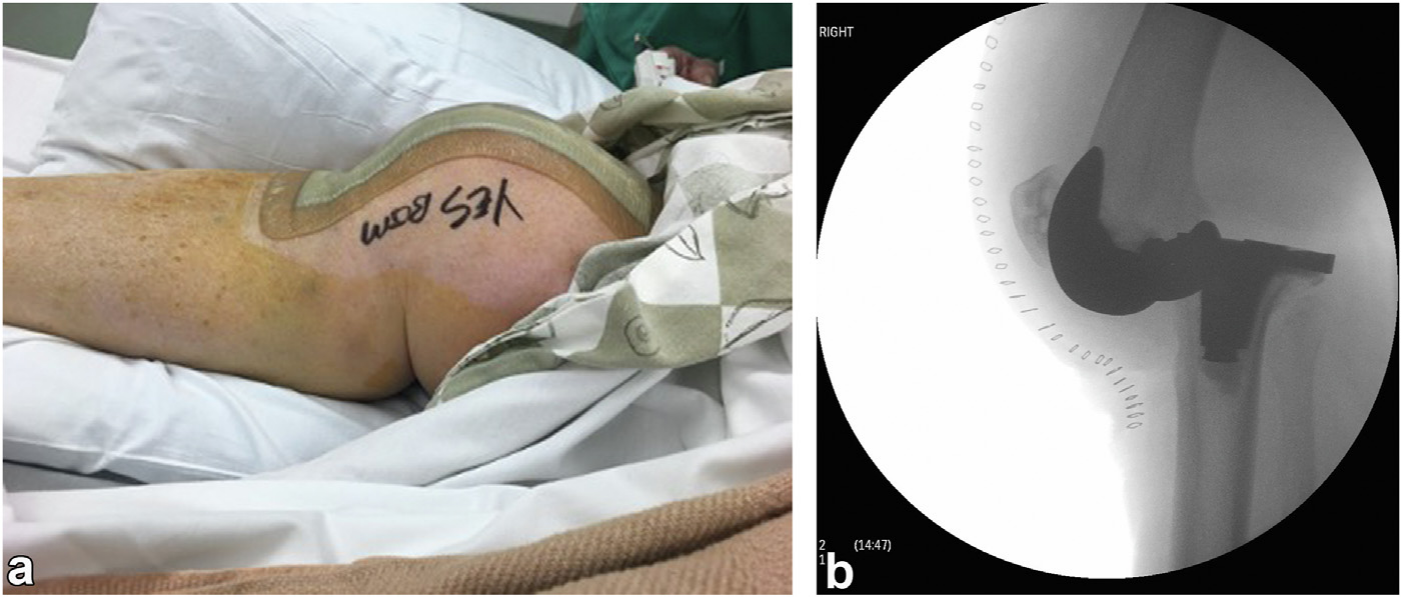
Posterior tibial dislocation following index surgery on (a) gross appearance and (b) fluoroscopy.

On the morning of POD 2, the knee was observed to be dislocated (Fig. 2a) and could not be reduced. There was no evidence of compartment syndrome, and distal pulses were intact and symmetrical with the other foot. She could activate the quadriceps, but there was marked medial and lateral hamstring spasm. Ankle dorsiflexion, plantar flexion, and extensor hallucis muscles were all functioning. There was no sensory deficit distally. She was taken to the operating room for closed reduction under anesthesia within 6 hours; the patient had eaten breakfast earlier in the morning, necessitating surgical delay for concern of her anesthesia status. Fluoroscopy confirmed posterior tibial dislocation (Fig. 2b). Closed reduction was performed successfully; the tibia had appeared posteriorly unstable in mid-flexion prior to paralysis under anesthesia, so a knee immobilizer in full extension was placed after her knee was reduced and with expectation that the instability and spasm may return as the anesthesia paralytic wore off. She was taken to recovery. Her knee was found to have dislocated again later that evening upon postoperative check, despite being immobilized in full extension.

### Revision right TKA, hamstring chemodenervation

On hospital day (HD) 3, the patient was taken to the operating room (OR) for a revision. Options for revision, including the removal of the current implant and implantation of a more constrained (eg, constrained condylar or hinged device) were discussed with the patient. Ultimately, revision of the polyethylene was decided upon after the shared decision-making discussion with the patient and her husband. Given her frailty and difficulty with pain control, we felt complete revision posed a significant likelihood of increased bone loss, blood loss, possible morbidity, and prolonged recovery. Polyethylene upsizing was considered the least invasive revision that could allow her to achieve adequate stability and function. Because her Parkinson's symptoms were mild at this time, it was hypothesized that her spasm was an acute response to the stress of surgery that would resolve, not a longstanding disease manifestation of neuromuscular disease status. Further, with her initial dislocation, we felt that her posterior/medial capsule was stripped from her tibia and femur and that if stability could be maintained until healing of the capsule was complete, then her instability would resolve permanently.

**Figure 3:**
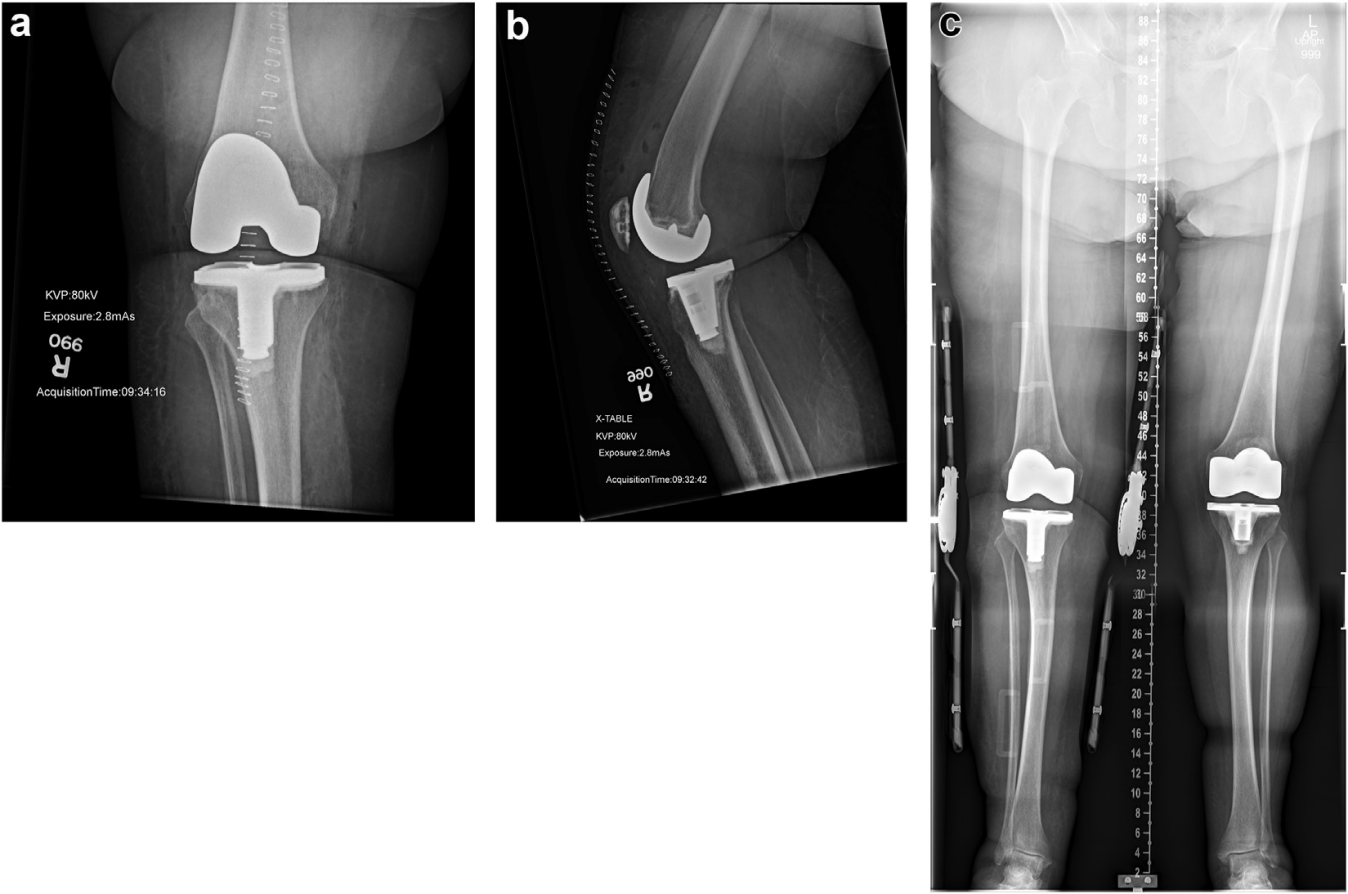
First right total knee arthroplasty (TKA) revision, immediately postoperatively. (a) AP and (b) lateral radiographic views demonstrate satisfactory alignment. Postoperative posterior slope measured at 6.2 degrees, clinically unchanged from preoperative measurement of 6.1 mm. Longstanding AP view of both legs at office follow-up (c).

Intraoperatively, there was no evidence of malposition, rupture of the extensor mechanism, or collateral ligament insufficiency. There was, however, mid-flexion instability of the tibia posteriorly and evidence that the posterior capsule and soft tissue sleeve had been stripped from the posteromedial femur and tibia. The knee was stable in 90 degrees of flexion and full extension, but in mid-flexion the tibia subluxated posteriorly and would dislocate when the patient exhibited a cough reflex under sedation. A 14-mm trial liner was placed, and the knee fully ranged. Stability was achieved throughout the arc of motion. Hamstring releases were considered but deemed unnecessary intraoperatively because the knee was balanced at the time of surgery with bony cuts and capsular injury. Postoperative anteroposterior and lateral radiographic views were satisfactory (Fig. 3). Of note, the patient’s posterior tibial slope (which may contribute to instability if excessive) was noted to be unchanged compared with preoperative measurement.

On HD 4, she was transitioned from a knee immobilizer to a hinged knee brace, locked in full extension. This was applied by PT without incident. She was able to take 3 small steps forward/back, limited by fatigue but without pain. After her PT session, the patient was assisted to bedside commode by nursing staff, when she then experienced a “pop” in her right knee. Recurrent posterior dislocation of her right knee was diagnosed, and she returned to the OR later that evening for reduction under general anesthesia as closed reduced at the bedside was not possible due to significant muscle spasm and patient discomfort. Under anesthesia, the knee was reduced with muscle relaxation, and the patient again had full ROM and excellent stability throughout the arc of motion. Revision to a more constrained or hinged model was again considered and had been discussed with the patient, but reassuring intraoperative findings of ROM and stability suggested that the current implant could be tolerated if protected from the forces of muscle spasm and the posterior capsule and soft tissue sleeve allowed to heal. A well-padded long leg splint was placed with sterile compressive dressing anteriorly. Patient was then placed on bedrest for 2 days to help prevent recurrent dislocation. Deep vein thrombosis prophylaxis consisted of aspirin 81 mg twice daily, along with thrombo-embolic deterrent stockings and Venodyne venous compression system prior to bedrest. At onset of bedrest, deep vein thrombosis prophylaxis medication was switched to enoxaparin 40 mg QD.

On HD 7, PT and occupational therapy performed bed-level reevaluations and initiated a slide board transfer from bed to a Barton Chair. She tolerated this well and remained hemodynamically stable. A knee immobilizer was worn on top of hard splint to enhance stability. For the next 5 days, activity orders were for bed to convertible stretcher chair via slide board only, 2-3 times daily.

A physiatrist (T.S.) was consulted for evaluation and consideration of BoNTA injection to the hamstrings to prevent recurrent dislocation. He felt the clinical picture confirmed suspicion that activation of the semitendinosus and long head biceps femoris were resulting in these unusual, recurrent dislocations. Asymmetric posterior force on the proximal tibia, with involuntary activation of the hamstrings, exacerbated by the dystonic effects of PD could be the etiology of the posterior knee dislocations. Chemodenervation was performed with 200 units of Onabotulinum toxin A (Botox, Allergan, Dublin, IRE) injected using electromyography guidance. The BoNTA was divided between 4 injection sites: 100 units were divided in 2 sites in the long head of the biceps femoris, and 100 units were divided between 2 semitendinosus sites. The expectation was that there would be an effect in 2-3 days, but it could take up to 2 weeks to achieve full desired efficacy. The injections were expected to last up to 3 months [6,7].

The patient remained on bedrest with bed to Barton chair privileges for 3 more days. On HD 12, her activity orders were upgraded to allow the patient to transfer out of bed and to weight bear as tolerated on her right lower extremity. She was able to ambulate 10 feet with assistance.

For the next 2 HDs, she was able to progress with PT/occupational therapy in her distance ambulated and ability to perform activities of daily living. Prior to discharge, her splint was removed, and she was transitioned back into her hinged knee brace (locked in full extension). The front portion of the fiberglass splint was positioned on top of hinged knee brace (fastened under 2 Velcro straps) and secured further with 2 elastic bandage wraps to ensure full extension. She was instructed to wear the second splint when out of bed, with only her hinged knee brace required when in bed.

On HD 14, she was discharged to an acute rehabilitation facility but decided to return home after just 1 day.

She returned at 2 weeks for staple removal, and gentle flexion ROM in her brace was initiated. Radiographs confirmed that the knee was located and in satisfactory alignment. By week 8, she demonstrated full extension to 105 degrees of flexion and had no instability. Her PD was under excellent control, and she was weaning from her cane.

At 6 months, the patient had a complete recovery with no pain, the ability to climb stairs, no use of a gait aid, and ROM of near full extension to 120 degrees of flexion. She had no instability of her knee and a Knee Society Knee Score (KSKS) (9) of 99. She did not pursue subsequent chemodenervation to the hamstrings due to adequate symptom control.

### Second revision right TKA, hamstring chemodenervation

Almost exactly 1 year after the initial right TKA operation, the patient sustained an unprovoked posterior TKA dislocation, for which she sought care at an outside emergency department. A closed reduction was performed at that time, and the patient returned home and subsequently reported 3 additional episodes of subluxation over the period of 1 week. The patient’s dislocation made it clear that healing the capsule without increasing constraint had not resolved her instability. This was reinforced by the fact that her PD had advanced. She presented to our emergency department for definitive management after another posterior tibial dislocation and was offered secondary revision surgery.

The patient underwent isolated polyethylene revision of her PS cemented TKA (NexGen, Zimmer Biomet, Warsaw, IN) 2 days after admission using a medial parapatellar approach. The Zimmer NexGen PS polyethylene was removed, and a condylar constrained knee (CCK) polyethylene was utilized in so-called “off-label” manner for increased stability; the polyethylene was revised from a size 14 posterior cruciate substituting to a size 17 for use with a size C femur and a size 2 tibia, with a polyethylene tibial locking bolt. This non-FDA-approved usage of a constrained polyethylene was discussed and agreed upon by the patient and her family preoperatively. The possibility of requiring revision to the femoral component and/or the usage of a hinged replacement was discussed, but given the patient’s frail state, removing a well-fixed implant would have definite morbidity; further, a review of the literature demonstrated that the complication rate for a rotating hinge type knee was 9%-63% [8]. Further revision was deemed unnecessary intraoperatively because the CCK-style polyethylene allowed for excellent stability. Spinal anesthesia was administered, and a postoperative adductor canal nerve block was performed utilizing ultrasound guidance.

A well-padded long leg splint was applied with a sterile compressive dressing and remained in place until the patient could undergo hamstring chemodenervation. The patient’s postoperative pain was again difficult to control.

On POD 3, the patient underwent BoNTA injection as a precautionary measure during the healing process. Given the effectiveness of the previous chemodenervation protocol, 400 units were again divided across 4 injection sites: 100 units were divided in 2 sites in the long head of the biceps femoris, and 100 units were divided between 2 semitendinosus sites. Following this procedure, she transitioned from a knee immobilizer to a hinged knee brace, unlocked with weight bearing as tolerated. The patient tolerated right knee ROM of 0-50 degrees of flexion and was able to walk 10 feet twice with assistance before she was discharged to acute rehabilitation on POD 3.

She remained in a hinged knee brace at all times until 3-week follow-up, at which point her knee appeared well-healed and stable, with ROM of near full extension to 100 degrees of flexion. She was advised to discontinue the mobilizer at that time and was subsequently cleared to start structured physical therapy 6 weeks postoperatively.

At 6 months, the patient was mobile with her cane and demonstrated ROM of full extension to 115 degrees of flexion.

At 1 year, the patient reported recovery with no knee pain; she walked with a walker, her baseline, with a ROM of full extension to 115 degrees of flexion. She had no instability of her knee and a KSKS [9] of 98. Her PD had progressed, and other chronic pain symptoms and difficulty balancing unrelated to the TKA significantly limited her mobility.

**Figure 4:**
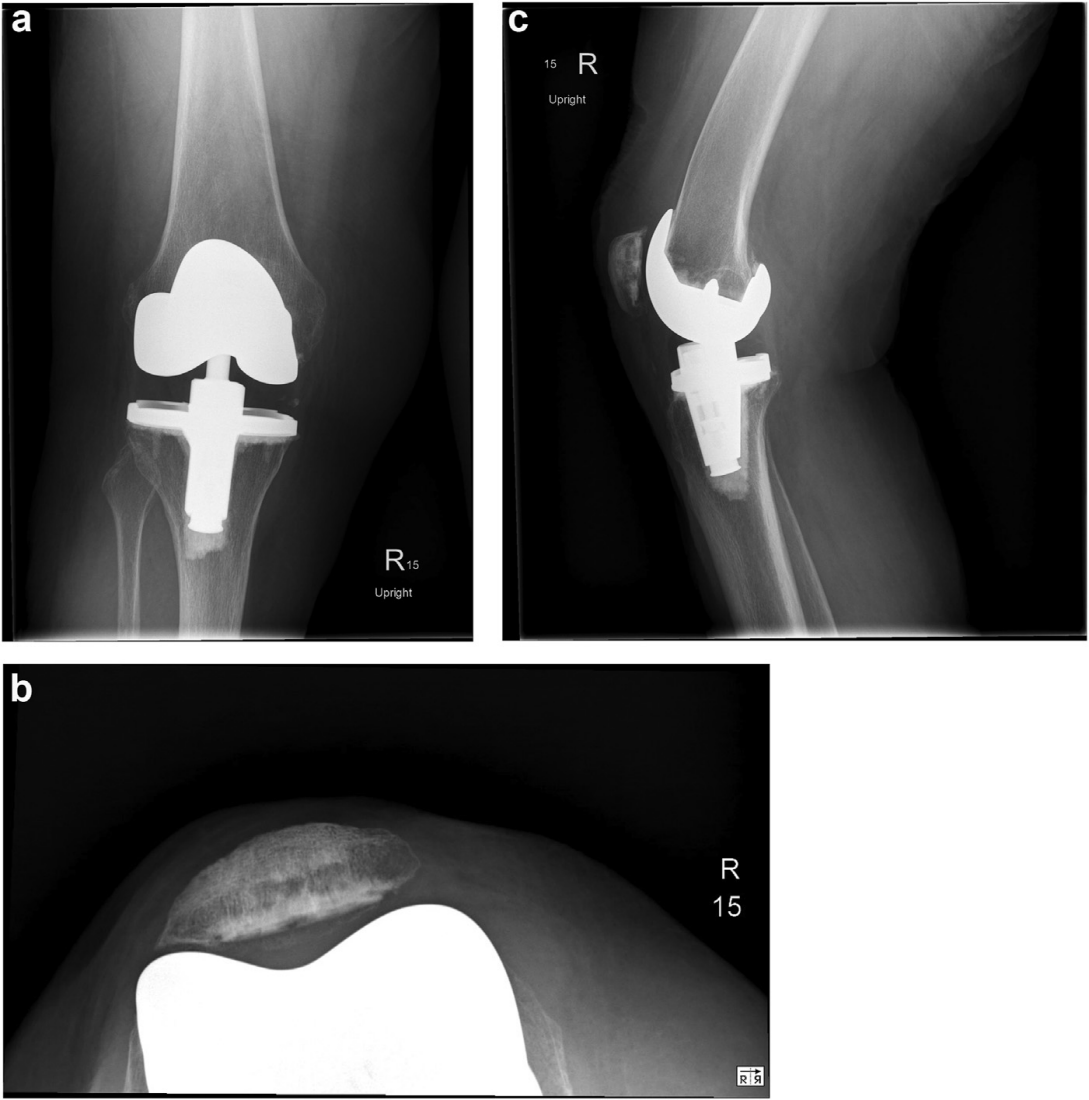
Second TKA revision at 2-year follow-up. (a) AP, (b) Merchant, and (c) lateral radiographic views demonstrate satisfactory alignment.

At 2 years after her last revision, the patient has had no instability and has no knee pain. She walks with a walker and exhibits a 15-degree flexion contracture with further flexion to 130 degrees and no instability. She has a KSKS (9) of 95 and a University of California, Los Angeles (UCLA) activity score of 3.5 [10]. Radiographs show no evidence of loosening or osteolysis (Fig. 4). The patient is satisfied with her care. Her PD has progressed significantly (now documented as modified Hoehn and Yahr [5] stage 3), and she presents with severe dyskinesia in the form of significant tremor and Pisa syndrome (lateral bending of the trunk). Symptoms unrelated to the TKA continue to limit her mobility. Follow-up is scheduled for 5 years after her recent revision. Of note, other than chemodenervation at the time of re-revision, she has not had further knee BoNTA injections.

## Discussion

Posterior knee instability after TKA in patients with neurodegenerative disorders has been previously described [1,[11], [12], [13], [14]], but the specific prevalence is not known. In the review by Macauley et al., [1] the authors show radiographs of 2 patients with early posterior instability and hypothesize that a weakened extensor mechanism or a hyper-rigid hamstring muscle group also may have contributed to the instability. They also reference a prior study with instability as a known complication [11]. They caution against the use of PS primary TKA in patients with severe PD, defined as a modified Hoehn and Yahr [5] score of 3 or more, and suggest the use of highly constrained hinged components in this patient population.

Minimal consensus has been reached in the literature regarding the overall benefits of TKA in patients with PD. Some studies suggest limited additional risk with similar functional outcomes to a matched control group without PD [15,16] while others report the opposite, with significantly worse functional outcomes compared to controls [17,18]. The literature supports an increased risk of complications and readmission in TKA in patients with PD [4,[19], [20], [21], [22]], with a meta-analysis by Min et al. [23] indicating PD patients had a 42% higher risk for any medical complication (*P* = .004) and a 65% higher risk for any surgical complication (*P* = .01) compared to a matched cohort. Consensus on the impact of TKA on quality of life (QoL) in this patient population is mixed and difficult to generalize due to the clinical variability in the PD course. Zong et al. [24] report that TKA has no benefit on QoL beyond a slight improvement in pain-related disability in the knee osteoarthritis patients with PD. Others report that TKA successfully provided pain relief in PD patients but did not improve functional ability [18,25]. Goh et al. [2] published similar findings, reporting that while patients with PD had relatively poorer knee function and QoL than matched controls, they still experienced significant functional gains compared with their preoperative status and high satisfaction. Functional outcome is related to disease progression and, therefore, widely variable and case-dependent.

We present the case of a patient with well-controlled moderate (modified Hoehn and Yahr [5] stage 2.5) PD who sustained acute, recurrent TKA posterior dislocations, recalcitrant to polyethylene revision and upsizing, after a PS TKA. PS TKA is not contraindicated and can work well in patients with mild PD and relatively normal quadriceps and hamstring function [1]. However, PS TKA is advised against in PD patients with severe disease in favor of cruciate-retaining, constrained condylar, or hinged devices [1]. The patient had a cruciate-retaining TKA on her contralateral side prior to presentation, a construct that likely prevented posterior subluxation; however, this construct was not appropriate for her subsequent TKA due to the advanced state of her osteoarthritis. The patient also had a flexion contracture present preoperatively, a prognostic factor indicating greater likelihood of postoperative flexion contracture; however, we are not aware of any studies that specifically address the impact of preoperative flexion deformity in TKA candidates with neuromuscular diseases [26]. Given the patient’s well-controlled disease, flexion contracture, and enough deformity that a cruciate-retaining implant was not appropriate, a PS-style TKA was utilized.

Balance in flexion and extension was tested thoroughly at this time of surgery, so flexion/extension gap asymmetry did not seem to be the cause of the instability. We therefore hypothesize that her instability was due to hamstring spasm and would like to expand the caution raised in the Macaulay et al. [1] review to include PD patients with less-severe disease. In these patients, usage of a cruciate-saving, constrained, or linked implant should be considered, as well as revision with on-label components, as surgery may temporarily lead to an increase in hamstring spasm. Contemporary primary total knee systems now offer higher levels of constraint that can be used routinely (eg, Persona Constrained Posterior Stabilized (CPS), Zimmer Biomet, Warsaw, IN). In addition, our case demonstrates that the use of BoNTA perioperatively could be considered, especially if posterior instability or severe spasms occur. It is unclear if modern ultracongruent or medial pivot-style implants can be used safely in this patient population.

The initial revision failed to give lasting stability in this case; full revision to a CCK polyethylene implant initially may have prevented return to the OR 1-year later. At the time of the initial revision, flexion and extension balance were equal, and removal of a recently cemented implant seemed excessive, so polyethylene upsizing was attempted. We had not discussed “off-label” usage of a CCK polyethylene prior to surgery, but this in retrospect would have been wise. Recent work by Sapountzis et al. [27] evaluated the rates of re-revision following TKA revision for symptomatic instability among patients who received either replacement of multiple components or isolated polyethylene liner exchange and found no statistical difference between component and liner exchange cohorts. However, this study noted a significantly lower rate of re-revision (12%) in cases where the level of constraint increased during component revision, a finding consistent with our experience [27].

Injection of intramuscular botulinum toxin has become available as a therapeutic option to treat various disorders of diffuse or focal cerebral and spinal conditions that lead to an increase in muscle tone, including PD [28]. Originally developed for treatment of strabismus and neurologic movement disorders, both new botulinum neurotoxin products and also wider indications have been established over the past decade [29]. Reports exist of BoNTA utilized to treat knee flexion contracture in PD patients following TKA [30,31] and complimentary to revision surgery to a more constrained construct secondary to posterior dislocation in a patient with another neurodegenerative condition, multiple sclerosis [32]. In our case, we used BoNTA in this “off-label” application after a shared decision-making discussion with the patient and her family. The involuntary activation of the hamstrings, exacerbated by the dystonic effects of PD, was thought to be a causative factor in her recurrent posterior dislocations. Our hypothesis was that chemodenervation of the semitendinosus and long head biceps femoris would relieve these muscle spasms and allow the knee to heal in proper alignment under a greater ROM. We thought that the soft tissue envelope stripping noted at the first revision surgery, if allowed to heal, might be a permanent restraint to dislocation. We thought that a single course of intramuscular BoNTA with appropriate immobilization would allow that healing. Results supported this hypothesis and demonstrated value in her postoperative BoNTA injection in the context of muscle spasm and neurodegenerative dystonia. However, the subsequent recurrence of posterior tibial instability vis-à-vis progression of PD symptoms highlights the complexity of total joint replacement in patients with neurodegenerative disease. We feel that when primary total knee replacement is indicated in a patient with PD, a cruciate retaining implant should be used in the case of minimal deformity, and a constrained or semiconstrained PS implant should be used in patients with advanced deformity or disease. With the benefit of hindsight and knowledge of the complex postoperative course, our patient would likely have had faster recovery and fewer procedures if a more constrained device had been used. Botulinum toxin injections may be used at the surgeon’s discretion in the perioperative period but are not useful for promoting long-term knee stability. It remains to be seen if ongoing, periodic injections would achieve this goal.

### Current controversies and future considerations

Although efficacy of TKA in patients with PD has yet to be conclusively established, many patients with knee arthritis and PD expect consideration of TKA when they have knee pain, stiffness, and associated dysfunction. Appropriate outcome measures and analysis should be sought to conclusively determine if improvement after TKA occurs in this cohort and in what specific aspects of the QoL spectrum. This information will be invaluable so that balanced and accurate shared decision-making may be offered.

Next, the role of BoNTA in this patient population needs further study. At a minimum, clinical research should be undertaken to determine if BoNTA is helpful in the treatment of knee arthritis. Further, the use of BoNTA should be elucidated in perioperative and/or postoperative management of primary and revision knee arthroplasty patients with varying stages of PD. We postulate that a program of gentle, sustained hamstring stretches, repeat BoNTA treatments, and possibly dynamic bracing might also have been effective, but certainly more taxing to the patient in terms of travel time, additional office visits, cost of PT and other copays, and time spent stretching (although time spent stretching is never really wasted). Theoretically, a permanent chemodenervation procedure could have also been performed using phenol motor point blocks, which are effectively an irreversible motor point chemical ablation [33]. Like the use of a constrained device, these options warrant consideration in PD patients presenting with instability following TKA.

Newer implant designs, specifically more constrained PS designs, blur the historic teaching that opined that a high level of natural or implant constraint is needed when considering knee replacement in patients with advanced PD. Future clinical research may be necessary to determine if the higher level of constraint used in newer primary TKA systems like the Zimmer Persona (CPS polyethylene, Zimmer Biomet, Warsaw, IN, USA) is adequate for PD patients. Currently, such constraint is suggested in correction of marked valgus deformity in patients with prior high tibial osteotomy, patellectomy, and revision surgeries.

One new finding from this case report that needs further examination is the role of implant design, specifically constraint, in patients with mild or well-controlled PD symptoms. It may be controversial to use higher levels of constraint in all patients with PD that undergo TKA, but this case example shows how a patient with less severe PD was susceptible to postoperative instability, and such patients may benefit from increased constraint.

## Summary

We present the case of a 77-year-old woman with well-controlled moderate (modified Hoehn and Yahr [5] stage 2.5) PD who sustained acute, recurrent TKA posterior dislocations, recalcitrant to polyethylene revision and upsizing, after a cruciate-sacrificing TKA. Temporary stability was obtained for a period of 1 year after postoperative hamstring injection with BoNTA and short-term immobilization, but 1-year postrevision, the patient again presented with acute, recurrent TKA posterior dislocations. She underwent a second polyethylene revision with increasing constraint, followed by hamstring injection with BoNTA and short-term postoperative immobilization. Successful stability was obtained and has been monitored over a period of 2 years. To our knowledge, this is the first report demonstrating that BoNTA can be used for adjuvant treatment of TKA instability but that increased constraint is necessary for longer-term instability resolution.

**KEY POINTS**

• Posterior total knee dislocation is a known complication associated with Parkinson’s disease (PD), thought to be due to contractures and spasms of the hamstring muscles.
• Although traditionally considered to most likely occur in PD patients with advanced neurodegenerative symptoms, we present a case of posterior total knee arthroplasty instability in a patient with well-controlled symptoms at index surgery.
• Use of a posterior cruciate-saving knee replacement design or either a linked or highly constrained posterior stabilized design is recommended in PD patients to minimize the chance of dislocation.
• Botulinum toxin A may be injected perioperatively into the hamstring muscles to mitigate spasm and allow healing, but it did not demonstrate a long-term treatment for instability in this case.

## Conflicts of interest

B. J. McGrory receives royalties from Smith and Nephew, Innomed, and Springer; is a speaker bureau of Smith and Nephew; and receives research support from Smith and Nephew. He receives royalties from Springer and serves as an editorial/governing board member of American Association of Hip and Knee Surgeons; all other authors declare no potential conflicts of interest.

For full disclosure statements refer to https://doi.org/10.1016/j.artd.2023.101273.

## Informed patient consent

The author(s) confirm that written informed consent has been obtained from the involved patient(s) or if appropriate from the parent, guardian, power of attorney of the involved patient(s); and, they have given approval for this information to be published in this case report (series).

## Appendix A Supplementary data

Conflict of Interest Statement for all the authors
