# Supplementary material for: Treatment of Postoperative Instability Following Total Knee Arthroplasty in Patients With Parkinson’s Disease
Source: Arthroplast Today. 2023 Dec 28;25:101273. doi: 10.1016/j.artd.2023.101273 (PMC10790002; doi:10.1016/j.artd.2023.101273)
Supplement: Conflict of Interest Statement for all the authors [file mmc1.pdf]

CONFLICT OF INTEREST STATEMENT

American Association of Hip and Knee Surgeons

(Adopted from the American Academy of Orthopaedic Surgeons disclosure statement)

The following form **must be filled out completely and submitted by each author (example, 6 authors, 6 forms). All items require a response. If there is no relevant disclosure for a given item, enter "*None*."**

Treatment of post-operative instability following total knee arthroplasty in patients with Parkinson's Disease

Manuscript Title

1. Royalties from a company or supplier (The following conflicts were disclosed)

   None to disclose

2. Speakers bureau/paid presentations for a company or supplier (The following conflicts were disclosed)

   None to disclose

3A. Paid employee for a company or supplier (The following conflicts were disclosed)

   None to disclose

3B. Paid consultant for a company or supplier (The following conflicts were disclosed)

   None to disclose

3C. Unpaid consultants for a company or supplier (The following conflicts were disclosed)

   None to disclose

4. Stock or stock options in a company or supplier (The following conflicts were disclosed)

   None to disclose

5. Research support from a company or supplier as a Principal Investigator (The following conflicts were disclosed)

   None to disclose

6. Other financial or material support from a company or supplier (The following conflicts were disclosed)

   None to disclose

7. Royalties, financial or material support from publishers (The following conflicts were disclosed)

   None to disclose

8. Medical/Orthopaedic publications editorial/governing board (The following conflicts were disclosed)

   None to disclose

9. Board member/committee appointments for a society (The following conflicts were disclosed)

   None to disclose

Each author must sign AND print or type his/her name, date and submit a separate form

In addition, one BLINDED Conflict of Interest form (no author names used) should be submitted per manuscript with all author disclosures.

THOMAS SAVADOVE, MD [signature] 6/13/2023

Author Name (Print or Type) Author Signature Date

CONFLICT OF INTEREST STATEMENT

American Association of Hip and Knee Surgeons

(Adopted from the American Academy of Orthopaedic Surgeons disclosure statement)

The following form **must be filled out completely and submitted by each author (example, 6 authors, 6 forms). All items require a response. If there is no relevant disclosure for a given item, enter "*None.*"**

Treatment of post operative instability following total knee arthroplasty in patients with Parkinson's Disease

Manuscript Title

1. Royalties from a company or supplier (The following conflicts were disclosed)

none

2. Speakers bureau/paid presentations for a company or supplier (The following conflicts were disclosed)

none

3A. Paid employee for a company or supplier (The following conflicts were disclosed)

none

3B. Paid consultant for a company or supplier (The following conflicts were disclosed)

none

3C. Unpaid consultants for a company or supplier (The following conflicts were disclosed)

none

4. Stock or stock options in a company or supplier (The following conflicts were disclosed)

none

5. Research support from a company or supplier as a Principal Investigator (The following conflicts were disclosed)

none

6. Other financial or material support from a company or supplier (The following conflicts were disclosed)

none

7. Royalties, financial or material support from publishers (The following conflicts were disclosed)

none

8. Medical/Orthopaedic publications editorial/governing board (The following conflicts were disclosed)

none

9. Board member/committee appointments for a society (The following conflicts were disclosed)

none

Each author must sign AND print or type his/her name, date and submit a separate form

In addition, one BLINDED Conflict of Interest form (no author names used) should be submitted per manuscript with all author disclosures.

LYDIA SOMMER	Lydia Sommer	4/24/23
Author Name (Print or Type)	Author Signature	Date

CONFLICT OF INTEREST STATEMENT

American Association of Hip and Knee Surgeons

(Adopted from the American Academy of Orthopaedic Surgeons disclosure statement)

The following form **must be filled out completely and submitted by each author (example, 6 authors, 6 forms). All items require a response. If there is no relevant disclosure for a given item, enter "*None.*"**

Treatment of post-operative instability following total knee arthroplasty in Patients with Parkinson's Disease

Manuscript Title

1. Royalties from a company or supplier (The following conflicts were disclosed)

   SMITH & NEPHEW, INNOMED, SPRINGER

2. Speakers bureau/paid presentations for a company or supplier (The following conflicts were disclosed)

   SMITH & NEPHEW

3A. Paid employee for a company or supplier (The following conflicts were disclosed)

   NA

3B. Paid consultant for a company or supplier (The following conflicts were disclosed)

   NA

3C. Unpaid consultants for a company or supplier (The following conflicts were disclosed)

   NA

4. Stock or stock options in a company or supplier (The following conflicts were disclosed)

   NA

5. Research support from a company or supplier as a Principal Investigator (The following conflicts were disclosed)

   SMITH & NEPHEW (INSTITUTIONAL)

6. Other financial or material support from a company or supplier (The following conflicts were disclosed)

   NA

7. Royalties, financial or material support from publishers (The following conflicts were disclosed)

   SPRINGER

8. Medical/Orthopaedic publications editorial/governing board (The following conflicts were disclosed)

   AAHKS (ARTHROPLASTY TODAY)

9. Board member/committee appointments for a society (The following conflicts were disclosed)

   NA

Each author must sign AND print or type his/her name, date and submit a separate form

In addition, one BLINDED Conflict of Interest form (no author names used) should be submitted per manuscript with all author disclosures.

BRIAN J. McGRORY	[signature]	5/30/2023
Author Name (Print or Type)	Author Signature	Date

CONFLICT OF INTEREST STATEMENT

American Association of Hip and Knee Surgeons

(Adopted from the American Academy of Orthopaedic Surgeons disclosure statement)

The following form **must be filled out completely and submitted by each author (example, 6 authors, 6 forms).**
All items require a response. If there is no relevant disclosure for a given item, enter "*None*."

Treatment of post-operative instability following total knee arthroplasty in patients with Parkinson's Disease
Manuscript Title

1. Royalties from a company or supplier (The following conflicts were disclosed)

   none

2. Speakers bureau/paid presentations for a company or supplier (The following conflicts were disclosed)

   none

3A. Paid employee for a company or supplier (The following conflicts were disclosed)

   none

3B. Paid consultant for a company or supplier (The following conflicts were disclosed)

   none

3C. Unpaid consultants for a company or supplier (The following conflicts were disclosed)

   none

4. Stock or stock options in a company or supplier (The following conflicts were disclosed)

   none

5. Research support from a company or supplier as a Principal Investigator (The following conflicts were disclosed)

   none

6. Other financial or material support from a company or supplier (The following conflicts were disclosed)

   none

7. Royalties, financial or material support from publishers (The following conflicts were disclosed)

   none

8. Medical/Orthopaedic publications editorial/governing board (The following conflicts were disclosed)

   none

9. Board member/committee appointments for a society (The following conflicts were disclosed)

   none

Each author must sign AND print or type his/her name, date and submit a separate form

In addition, one BLINDED Conflict of Interest form (no author names used) should be submitted per manuscript with all author disclosures.

Catherine Call	Catherine Call	6/12/23
Author Name (Print or Type)	Author Signature	Date

CONFLICT OF INTEREST STATEMENT

American Association of Hip and Knee Surgeons

(Adopted from the American Academy of Orthopaedic Surgeons disclosure statement)

The following form **must be filled out completely and submitted by each author (example, 6 authors, 6 forms). All items require a response. If there is no relevant disclosure for a given item, enter "*None*."**

Treatment of post-operative instability following total knee arthroplasty in patients with Parkinson's Disease

Manuscript Title

1. Royalties from a company or supplier (The following conflicts were disclosed)

   None

2. Speakers bureau/paid presentations for a company or supplier (The following conflicts were disclosed)

   None

3A. Paid employee for a company or supplier (The following conflicts were disclosed)

   None

3B. Paid consultant for a company or supplier (The following conflicts were disclosed)

   None

3C. Unpaid consultants for a company or supplier (The following conflicts were disclosed)

   None

4. Stock or stock options in a company or supplier (The following conflicts were disclosed)

   None

5. Research support from a company or supplier as a Principal Investigator (The following conflicts were disclosed)

   None

6. Other financial or material support from a company or supplier (The following conflicts were disclosed)

   None

7. Royalties, financial or material support from publishers (The following conflicts were disclosed)

   None

8. Medical/Orthopaedic publications editorial/governing board (The following conflicts were disclosed)

   None

9. Board member/committee appointments for a society (The following conflicts were disclosed)

   None

Each author must sign AND print or type his/her name, date and submit a separate form

In addition, one BLINDED Conflict of Interest form (no author names used) should be submitted per manuscript with all author disclosures.

Erica Thompson	[signature]	4/25/23
Author Name (Print or Type)	Author Signature	Date
